# Galanin receptor 3 attenuates inflammation and influences the gut microbiota in an experimental murine colitis model

**DOI:** 10.1038/s41598-020-79456-y

**Published:** 2021-01-12

**Authors:** Susanne M. Brunner, Florian Reichmann, Julia Leitner, Soraya Wölfl, Stefan Bereswill, Aitak Farzi, Anna-Maria Schneider, Eckhard Klieser, Daniel Neureiter, Michael Emberger, Markus M. Heimesaat, Daniel Weghuber, Roland Lang, Peter Holzer, Barbara Kofler

**Affiliations:** 1 Research Program for Receptor Biochemistry and Tumor Metabolism, Department of Pediatrics, University Hospital of the Paracelsus Medical University, Muellner Hauptstr. 48, 5020 Salzburg, Austria; 2 Research Unit of Translational Neurogastroenterology, Division of Pharmacology, Otto Loewi Research Center, Medical University of Graz, Universitätsplatz 4, 8010 Graz, Austria; 3 Laboratory for Pathology Weger, Emberger, Strubergasse 20, 5020 Salzburg, Austria; 4 Institute of Microbiology, Infectious Diseases and Immunology, Charité – University Medicine Berlin, Garystr. 5, 14195 Berlin, Germany; 5 Department of Pediatrics, University Hospital of the Paracelsus Medical University, Muellner Hauptstr. 48, 5020 Salzburg, Austria; 6 Institute of Pathology, University Hospital of the Paracelsus Medical University, Muellner Hauptstr. 48, 5020 Salzburg, Austria; 7 Department of Dermatology and Allergology, University Hospital of the Paracelsus Medical University, Muellner Hauptstr. 48, 5020 Salzburg, Austria

**Keywords:** Inflammatory bowel disease, Acute inflammation, Mucosal immunology

## Abstract

The regulatory (neuro)peptide galanin and its three receptors (GAL_1–3_R) are involved in immunity and inflammation. Galanin alleviated inflammatory bowel disease (IBD) in rats. However, studies on the galanin receptors involved are lacking. We aimed to determine galanin receptor expression in IBD patients and to evaluate if GAL_2_R and GAL_3_R contribute to murine colitis. Immunohistochemical analysis revealed that granulocytes in colon specimens of IBD patients (Crohn’s disease and ulcerative colitis) expressed GAL_2_R and GAL_3_R but not GAL_1_R. After colitis induction with 2% dextran sulfate sodium (DSS) for 7 days, mice lacking GAL_3_R (GAL_3_R-KO) lost more body weight, exhibited more severe colonic inflammation and aggravated histologic damage, with increased infiltration of neutrophils compared to wild-type animals. Loss of GAL_3_R resulted in higher local and systemic inflammatory cytokine/chemokine levels. Remarkably, colitis-associated changes to the intestinal microbiota, as assessed by quantitative culture-independent techniques, were most pronounced in GAL_3_R-KO mice, characterized by elevated numbers of enterobacteria and bifidobacteria. In contrast, GAL_2_R deletion did not influence the course of colitis. In conclusion, granulocyte GAL_2_R and GAL_3_R expression is related to IBD activity in humans, and DSS-induced colitis in mice is strongly affected by GAL_3_R loss. Consequently, GAL_3_R poses a novel therapeutic target for IBD.

## Introduction

Neurogenic and inflammatory factors interactively contribute to the pathogenesis of inflammatory bowel diseases (IBDs) like Crohn’s disease (CD) or ulcerative colitis (UC)^1–4^. Consequently, neuropeptides play a key role in modulating disease activity^3,4^. The regulatory (neuro)peptide galanin is widely distributed in the nervous system and is expressed by non-neuronal tissues. Galanin, a 29 amino acid peptide (30 aa in humans) derived from a larger precursor peptide (ppGAL) by proteolytic cleavage, mediates its effects via three G protein-coupled receptors (GAL_1–3_R)^5^. They differ in their distribution pattern, functional coupling and signaling pathways. GAL_1_R and GAL_3_R predominantly couple to G_i/o_, leading to a reduction of cAMP and inactivation of protein kinase A (PKA). GAL_2_R signals via multiple classes of G proteins, preferentially via G_q/11_, resulting in the activation of protein kinase C (PKC)^5^. PKA and PKC regulate immune cell functions^6,7^. Importantly, we and others observed expression of galanin and galanin receptors (GALRs) in different immune cells, including neutrophils, natural killer (NK) cells, monocytes, macrophages, B and T cells^8–12^. Accordingly, galanin potently modulates neutrophil, NK cell, monocyte and macrophage functions in vitro^8–11^. Furthermore, GAL_3_R is involved in inflammatory diseases in vivo, including experimental arthritis, psoriasis and pancreatitis^13–15^.

Galanin-like immunoreactivity is found in enteric nerve bodies and fibers in all layers of the gut wall and at all levels of the gastrointestinal tract (GIT)^16–20^ with increased expression under inflammatory conditions^21,22^. In the gut, galanin influences the release of neurohumoral substances, gut motility, smooth muscle contractility, fluid secretion and intestinal ion flux^3,23^. In the colon, galanin-specific binding sites, indicating presence of GALRs, were found in the myenteric plexus^24^, in smooth muscle cells and nerve fibers in the submucosal layer, but also in the lamina epithelialis mucosae lining the crypts^25^. The density of galanin-specific binding sites in the colon remained unaffected by IBD^24^. In the distal and proximal rat colon comparable GAL_1_R and GAL_2_R mRNA levels but low levels of GAL_3_R were found^26^. In contrast, only GAL_1_R mRNA was detected in colonic cell lines and in epithelial cells of human colon^27,28^. Weak positive immunohistochemical (IHC) staining of GAL_1_R was observed in only few epithelial cells, but not in crypts. Interestingly, patients with diverse inflammatory diseases affecting the colon exhibited dramatically increased GAL_1_R protein expression in colonic tissue, including crypts^27^. Importantly, in rats with trinitrobenzenesulfonic acid (TNBS)-induced colitis, GALR agonism with galanin ameliorated the extent and severity of the colonic injury and reduced myeloperoxidase (MPO) activity, TNFα levels and nitric oxide production^29,30^. In contrast, in dextran sodium sulfate (DSS)-induced colitis in mice, GALR inhibition with a non-selective GALR antagonist blunted colonic inflammation^22^. However, GAL_1_R loss only affected colonic fluid secretion^27,31–33^ but not disease activity^33^, indicating that GAL_1_R might not play a central role in IBD pathophysiology. Yet, the GALR subtype mediating galanin-related effects on IBD has not been identified. Furthermore, data on protein expression of GAL_2_R and GAL_3_R in human colon were missing due to the lack of specific antibodies until recently.

Consequently, we hypothesized that colonic GALR expression might be altered during colitis and that colonic inflammation might be affected by GAL_2_R or GAL_3_R signaling. In this study we aimed to characterize GALR expression in the colon of healthy subjects and patients with CD and UC by IHC using carefully validated antibodies^34^. Furthermore, we investigated the role of GAL_2_R and GAL_3_R in a mouse model of colitis by analyzing histologic damage to the colon and immune activation in wild-type (WT) and receptor knockout (KO) mice.

Moreover, there is growing awareness that the interplay between commensal bacteria and the innate immune response has a pivotal role in IBD^35^ and influences disease susceptibility^35,36^. Additionally, evidence has recently emerged that neuropeptides impact the gut microbiota^37^. Therefore, we hypothesized that during colitis the gut microbiota might be altered in mice lacking GAL_2_R or GAL_3_R. Thus, we aimed to determine the composition of the intestinal microbiota in experimental animals following colitis induction.

## Results

### Granulocytes in colon tissue of CD and UC patients express galanin receptors 2 and 3

**Figure 1:**
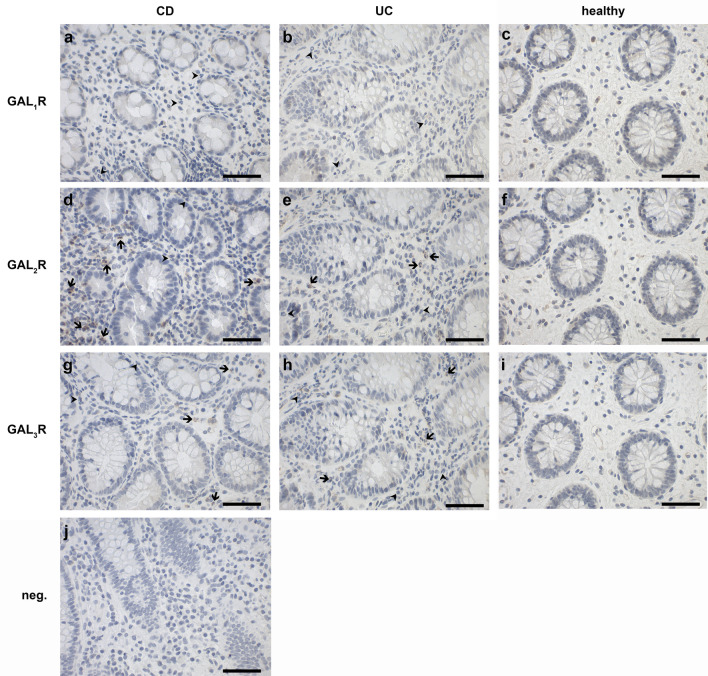
Representative images of IHC staining of human colon specimens of patients diagnosed with Crohn’s disease [CD; case 15 (**a**), case 16 (**d**), case 18 (**g**)] or ulcerative colitis [UC; case 25 (**b**, **e**, **h**)] and healthy individuals [case 9 (**c**, **f**, **i**)] for GAL_1_R (**a**–**c**), GAL_2_R (**d**–**f**) and GAL_3_R (**g**–**i**). Arrowheads indicate GALR-negative and arrows GALR-positive granulocytes. A negative control with second antibody only is shown (**j**). Scale bar: 50 µm.

As the galanin system is involved in inflammatory processes, we hypothesized that the expression of GALRs is altered in IBD patients. IHC analysis revealed that colonocytes, goblet cells and epithelial cells of the colonic mucosa were negative for GALR staining, independent of diagnosis (data not shown). Positive staining for GAL_3_R in colonic blood vessels was observed in 21 out of 24 subjects (Fig. S1), which was described previously in dermal vessels^14^. In the colonic mucosa of all subjects, single lymphocytes showing positive staining for all GALRs were found (data not shown). In IBD, active disease status is characterized by substantial infiltration by granulocytes into the mucosa. In our study cohort, 7 of 10 CD and 2 of 5 UC patients presented with active disease. Remarkably, weak to strong focal cytoplasmatic positivity for GAL_2_R and weak positivity for GAL_3_R but no positive staining for GAL_1_R was observed in mucosal granulocytes of all patients with active disease. Interestingly, only a subset of granulocytes found in inflamed human colon was positive for GAL_2_R (62.6% of neutrophils) or GAL_3_R (38.9% of neutrophils) immunoreactivity (Fig. 1; Table 1). In colon specimens of healthy individuals (Fig. 1c,f,i) or patients with inactive disease (data not shown), no or only very few granulocytes were found.

**Table 1:** Numbers (means ± SD in %) of GAL_2_R- and GAL_3_R-positive (GAL_2_R^+^ or GAL_3_R^+^) neutrophilic granulocytes in human colon specimen of patients diagnosed with Crohn’s disease (CD) or ulcerative colitis (UC).

Disease group	Disease status (n)	GAL_2_R^+^ (%)	GAL_3_R^+^ (%)
IBD	Active (9)	62.7 ± 32.3	38.9 ± 24.7
	Non-active (6)	16.7 ± 40.8	16.7 ± 40.8
	All (15)	44.3 ± 41.7	30.0 ± 32.7
CD	Active (7)	72.3 ± 29.6	42.5 ± 27.2
	Non-active (3)	0	0
	All (10)	50.6 ± 42.5	29.7 ± 30.2
UC	Active (2)	28.8 ± 11.4	26.3 ± 6.1
	Non-active (3)	33.3 ± 57.7	33.3 ± 57.7
	All (5)	31.5 ± 41.3	30.5 ± 41.1

### DSS-induced colitis is exacerbated in GAL_3_R-KO but not GAL_2_R-KO mice

Because we found GAL_2_R and GAL_3_R to be expressed on granulocytes in colonic mucosa of IBD patients, we studied the progression of DSS-induced colitis in mice lacking GAL_2_R or GAL_3_R. These mouse lines were on different genetic backgrounds, C57BL/6J and C57BL/6N, respectively. Therefore, the course of colitis in GAL_2_R-KO and GAL_3_R-KO mice was analyzed only in comparison to the corresponding WT group.

**Figure 2:**
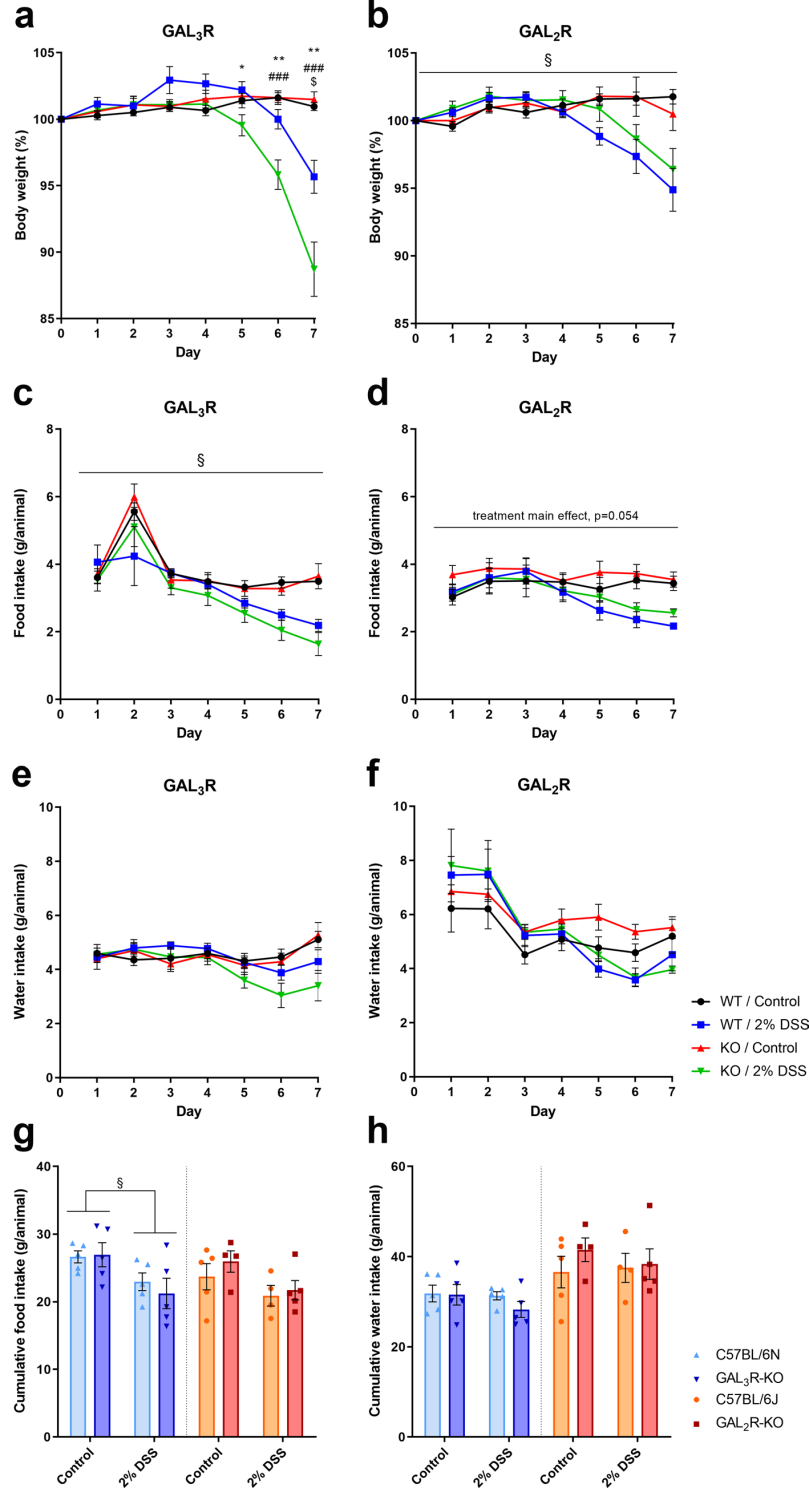
Body weight (% to starting weight) (**a**,**b**), food (**c**,**d**) and water intake (**e**,**f**) following colitis induction, as well as cumulative food (**g**) and water intake (**h**) in GAL_3_R-KO (**a**,**c**,**e**,**g**,**h**), GAL_2_R-KO (**b**,**d**,**f**,**g**,**h**) and corresponding WT mice. Data represent means ± SEM. n = 9–11. Data were analyzed by two-way RM or two-way ANOVA followed by appropriate post hoc tests. Main effect treatment, ^§^*p* < 0.05; WT/Ctl versus WT/DSS, ^$^*p* < 0.05; KO/Ctl versus KO/DSS, ^###^*p* < 0.001; WT/DSS versus KO/DSS, ^*^p < 0.05; ^**^p < 0.01.

The first evidence of an effect of GAL_2_R or GAL_3_R loss on colitis was revealed by changes in body weight following colitis induction with DSS. Interestingly, in GAL_3_R-KO and corresponding WT mice, two-way ANOVA analysis of body weight data showed a significant interaction between genotype and treatment on treatment days 4 to 7 (*p* < 0.05; detailed F statistics are shown in Supplementary Table S5). Compared to untreated control mice, DSS treatment resulted in progressive loss of body weight that reached significance on treatment day 7 in treated GAL_3_R-WT mice (*p* = 0.025) and on days 6 (*p* < 0.001) and 7 (*p* < 0.001) in DSS-treated GAL_3_R-KOs. Weights of control animals did not differ between genotypes. Remarkably, GAL_3_R-KOs with colitis lost significantly more body weight on treatment days 5, 6 and 7 than DSS-treated WT mice (*p* < 0.05). At day 7, GAL_3_R-WT mice had lost 4.3% of their starting body weight, whereas GAL_3_R-KOs had lost 11.3% (*p* = 0.002) (Fig. 2a). As expected, colitis induction resulted in reduced food consumption (main effect treatment, *p* = 0.011; Fig. 2g). Importantly, the exaggerated loss of body weight in GAL_3_R-KOs was not caused by a stronger reduction in food intake. DSS-treated GAL_3_R-WT and GAL_3_R-KO mice consumed less food on treatment days 5–7 compared to day 1 (*p* < 0.05), with no difference between genotypes. Untreated mice consumed more food on day 2, and beyond that, the food intake remained constant over the 7-days treatment period (Fig. 2c). Furthermore, as the daily and cumulative water intake was independent of genotype or treatment, we can exclude the possibility that increased consumption of DSS-containing water contributed to the greater loss of body weight in GAL_3_R-KOs (Fig. 2e,h).

In contrast, GAL_2_R-KO mice exhibited a similar reduction in body weight compared to GAL_2_R-WT mice in response to DSS over the 7-days treatment period (treatment main effect, *p* = 0.027). Compared to baseline, DSS-treated GAL_2_R-KO and GAL_2_R-WT mice weighed less on treatment day 7 (*p* = 0.030), while control mice gained weight which reached significance on days 2 (*p* = 0.048) and 5 (*p* = 0.009), independent of genotype (Fig. 2b). Daily food and water intake remained constant in untreated mice, with no difference between genotypes (Fig. 2d,f). GAL_2_R-KO and GAL_2_R-WT mice with colitis consumed less food on days 6 (*p* = 0.005) and 7 (*p* = 0.003) compared to day 1, independent of genotype (Fig. 2d). The cumulative food intake showed only a trend toward being reduced in DSS-treated mice compared to controls (main effect treatment, *p* = 0.054) (Fig. 2g). Daily water intake was reduced in DSS-treated mice on treatment days 5 (*p* = 0.029) and 6 (*p* = 0.011) compared to day 1, independent of GAL_2_R genotype (Fig. 2f). The cumulative water intake was unaffected by GAL_2_R genotype or treatment (Fig. 2h).

### Histologic damage and immune cell infiltration in DSS-induced colitis are exaggerated in GAL_3_R-KO mice

The increased loss of body weight in GAL_3_R-KO mice in response to DSS treatment indicated that colitis might develop more severely if GAL_3_R is lacking. Consequently, we analyzed disease-related variables, histologic and molecular parameters in colonic tissue and circulating cytokine/chemokine levels in experimental animals on treatment day 7 to further evaluate the course of inflammation in KO and WT mice.

**Figure 3:**
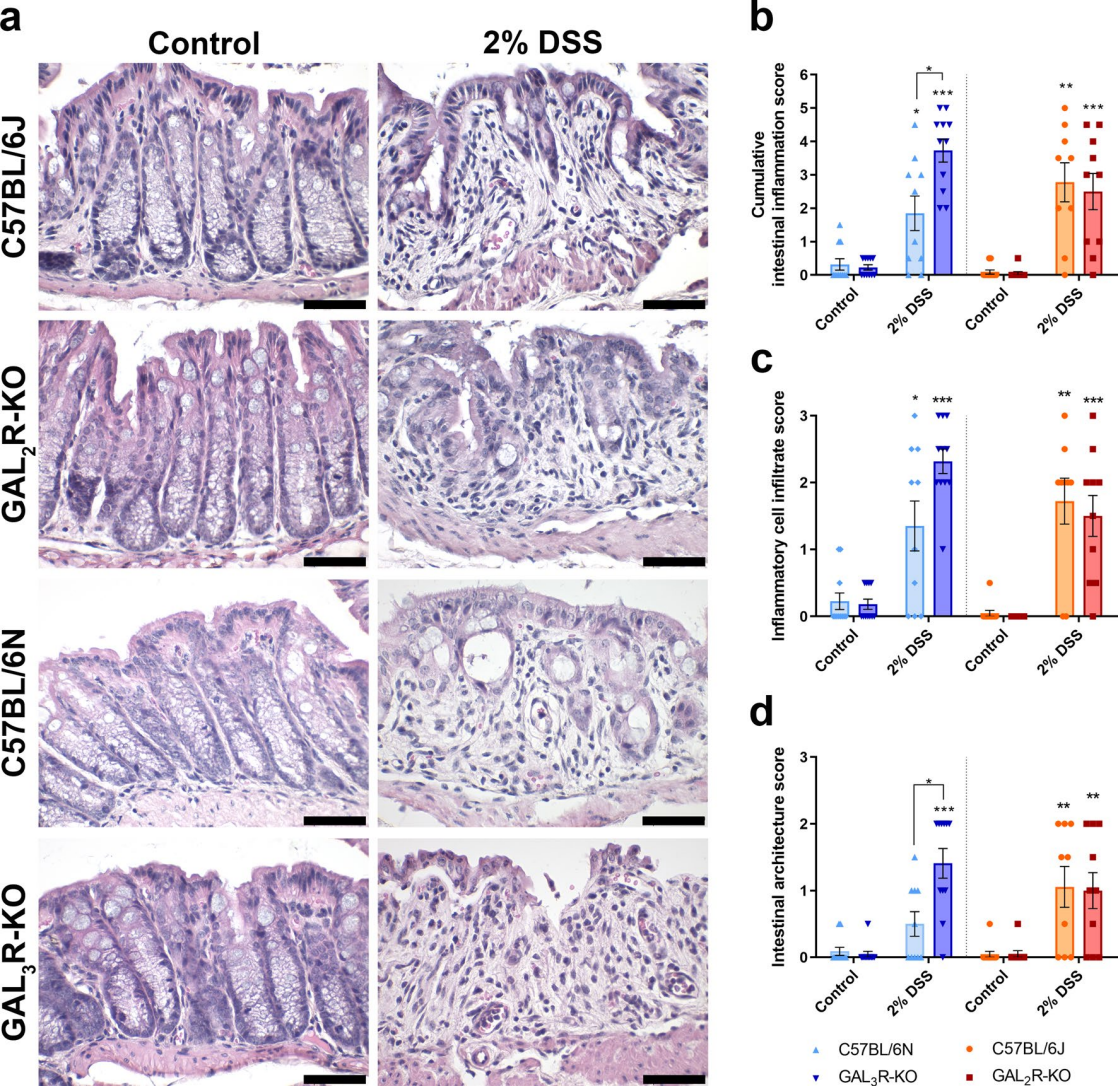
Intestinal inflammation in the distal colon of GAL_3_R-KO, GAL_2_R-KO and corresponding WT mice. Representative images of HE staining (Scale bar: 50 µm) (**a**). Cumulative semiquantitative scores of intestinal inflammation (**b**). Score points for inflammatory cell infiltrate (**c**). Score points for intestinal architecture (**d**). Data represent means ± SEM. n = 9–11. Data were analyzed by Kruskal–Wallis test followed by Mann–Whitney U test. *p < 0.05; **p < 0.01; ***p < 0.001 versus corresponding controls or as indicated.

Intestinal inflammation was more severe in DSS-treated GAL_3_R-KOs compared to GAL_3_R-WT mice. Histologically, the distal colon of GAL_3_R-KOs showed a trend toward more severe inflammatory cell infiltration during colitis compared to GAL_3_R-WT mice (*p* = 0.061), with transmural inflammation occurring in some animals (Fig. 3a,c). Importantly, GAL_3_R-KOs had barely preserved epithelial linings with extended ulcerations to the mucosa and destroyed crypts, while 60% of DSS-treated GAL_3_R-WT mice presented an intact epithelium or only minor changes to the epithelium and the mucosal architecture (Fig. 3a,d). Accordingly, histologic score points for damage to the mucosa were significantly higher in DSS-treated GAL_3_R-KOs compared to GAL_3_R-WT mice (*p* = 0.010) (Fig. 3d). Overall, the cumulative intestinal inflammation score was higher in DSS-treated GAL_3_R-KOs compared to GAL_3_R-WT mice (*p* = 0.016) (Fig. 3b).

The results relating to colitis obtained in GAL_2_R-KO mice were different from those observed in GAL_3_R-KO mice. Histologic evaluation of the distal colon revealed a similar mild to moderate infiltration of immune cells into the mucosa and submucosa in GAL_2_R-KO and GAL_2_R-WT mice following DSS treatment (Fig. 3a,c). While control mice showed undamaged epithelia and no changes to the mucosal architecture, DSS-treated GAL_2_R-KO and GAL_2_R-WT mice presented with (focal) erosions of the epithelium and destroyed crypts (Fig. 3a,d). Overall, the intestinal inflammation following colitis induction was similar in GAL_2_R-KO and WT animals (Fig. 3b).

The disease-related parameters colon weight, colon length, colon weight to length ratio, spleen weight and disease activity score (DAS) were significantly altered by DSS treatment in all groups (*p* < 0.05), except that GAL_3_R-KO and GAL_3_R-WT mice showed only a trend toward increased colon weight in response to DSS (*p* = 0.069). However, there were no significant differences in the disease-related parameters between DSS-treated GAL_3_R-KO and GAL_3_R-WT or between DSS-treated GAL_2_R-KO and GAL_2_R-WT mice (Fig. S2).

### Colonic MPO content and number of infiltrating neutrophils in DSS-induced colitis are higher in GAL_3_R-KO mice

**Figure 4:**
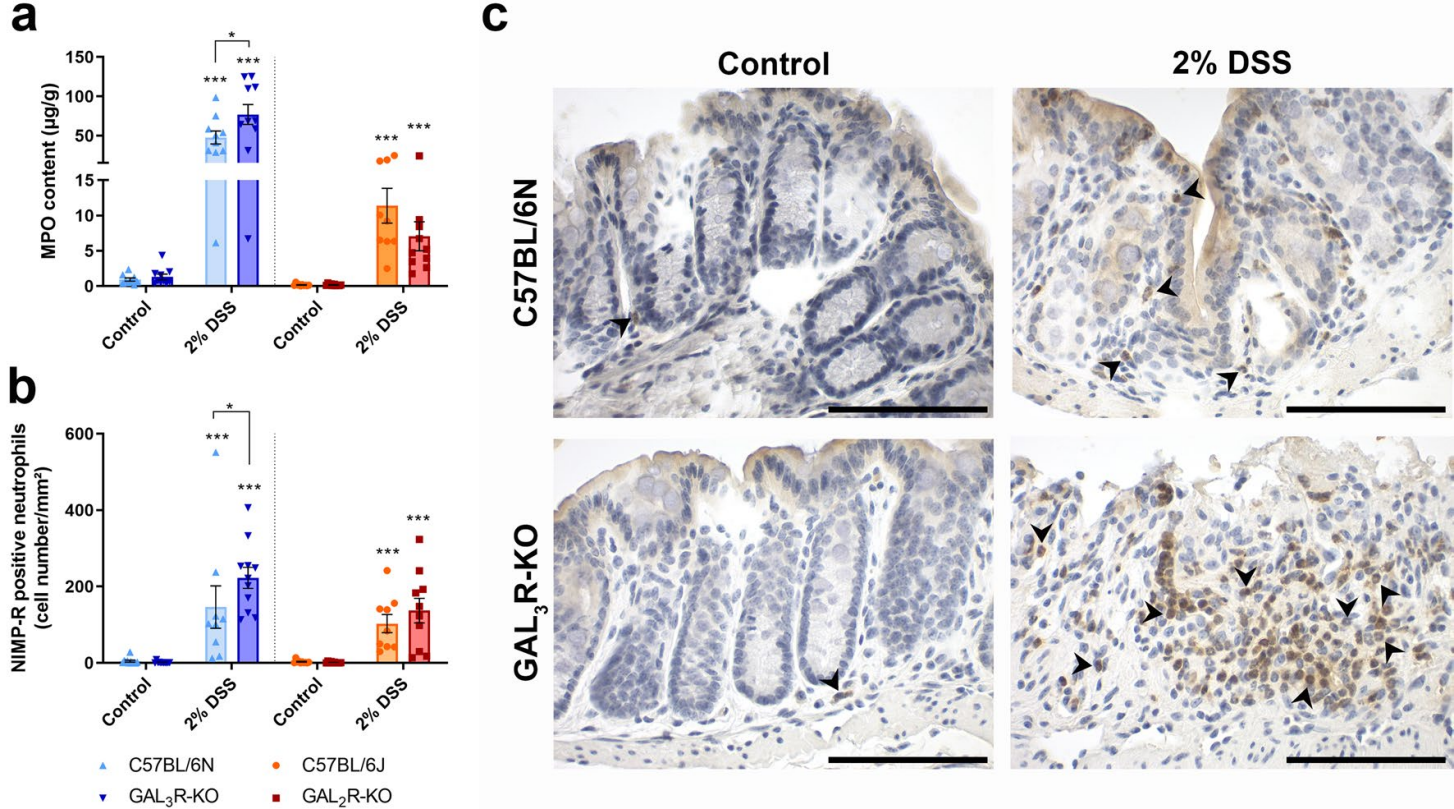
Infiltration of the colon by neutrophils in GAL_3_R-KO, GAL_2_R-KO and corresponding WT mice was evaluated by neutrophil-derived MPO content (**a**) and numbers of NIMP-R14^+^ neutrophils (**b**). Data represent means ± SEM. n = 9–11. Data were analyzed by Kruskal–Wallis test followed by Mann–Whitney-U test. *p < 0.05; ***p < 0.001 versus corresponding controls or as indicated. Representative images of IHC staining of murine colon samples against NIMP-R14 in C57BL/6N and GAL_3_R-KO mice (**c**). Arrowheads indicate NIMP-R14-positive neutrophils. Scale bar: 50 µm.

As semiquantitative scores indicated that accumulation of inflammatory cells in the colon tissue appeared to be increased in DSS-treated GAL_3_R-KO mice, we evaluated the amount of neutrophil-derived MPO in distal colon as an index of neutrophil influx. Colitis induction significantly elevated MPO levels in all DSS-treated groups compared to corresponding controls (*p* < 0.001). In agreement with the histomorphological evaluation, GAL_3_R-KOs had higher MPO levels compared to GAL_3_R-WT mice following DSS treatment (*p* = 0.041) (Fig. 4a). This finding is supported by an increased number of infiltrating NIMP-R14^+^ neutrophils in distal colon tissue of all DSS-treated mice compared to corresponding controls (*p* < 0.001). In addition, significantly more NIMP-R14^+^ neutrophils invaded the colon tissue of GAL_3_R-KOs compared to GAL_3_R-WT mice (*p* = 0.025) (Fig. 4b,c). In contrast, DSS-treated GAL_2_R-KO and GAL_2_R-WT animals exhibited a similar increase in MPO content and numbers of infiltrated neutrophils in the colon (Fig. 4a,b).

### Colonic and systemic levels of inflammatory cytokines and chemokines are modulated by GAL_3_R deletion

The aggravated intestinal inflammation in GAL_3_R-KO animals might be caused by altered cytokine/chemokine expression. Therefore, we analyzed pro- and anti-inflammatory cytokine and chemokine mRNA levels in colon tissue and protein levels in plasma.

**Figure 5:**
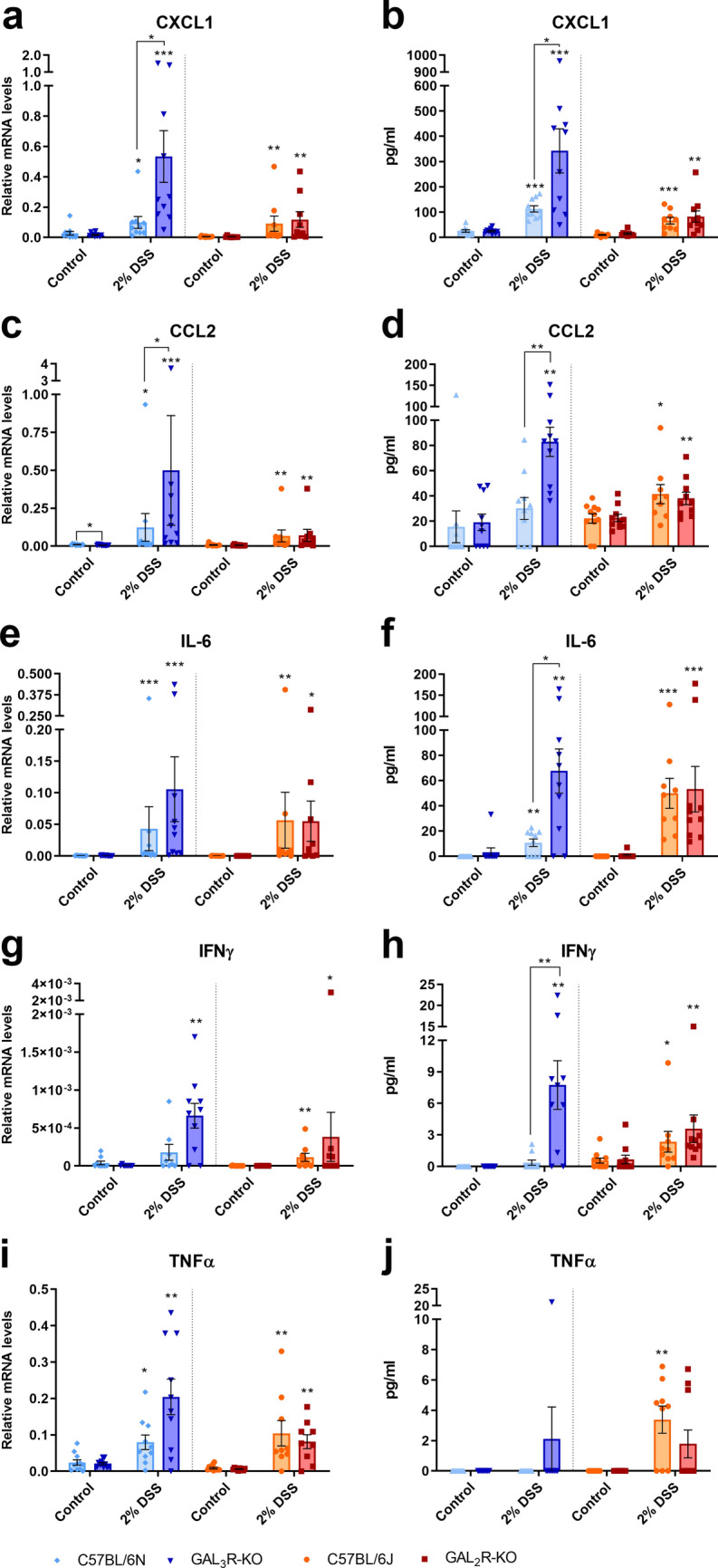
Relative mRNA expression levels (**a**,**c**,**e**,**g**,**i**) in the colon and plasma protein levels (**b**,**d**,**f**,**h**,**j**) of CXCL1 (**a**,**b**), CCL2 (**c**,**d**), IL-6 (**e**,**f**), IFNγ (**g**,**h**), and TNFα (**i**,**j**) in GAL_3_R-KO, GAL_2_R-KO and corresponding WT mice. Levels of mRNA were determined relative to the housekeeping gene HPRT. Data represent means ± SEM. n = 7–11. Data were analyzed by Kruskal–Wallis test followed by Mann–Whitney U test. *p < 0.05; **p < 0.01; ***p < 0.001 versus corresponding controls or as indicated.

**Figure 6:**
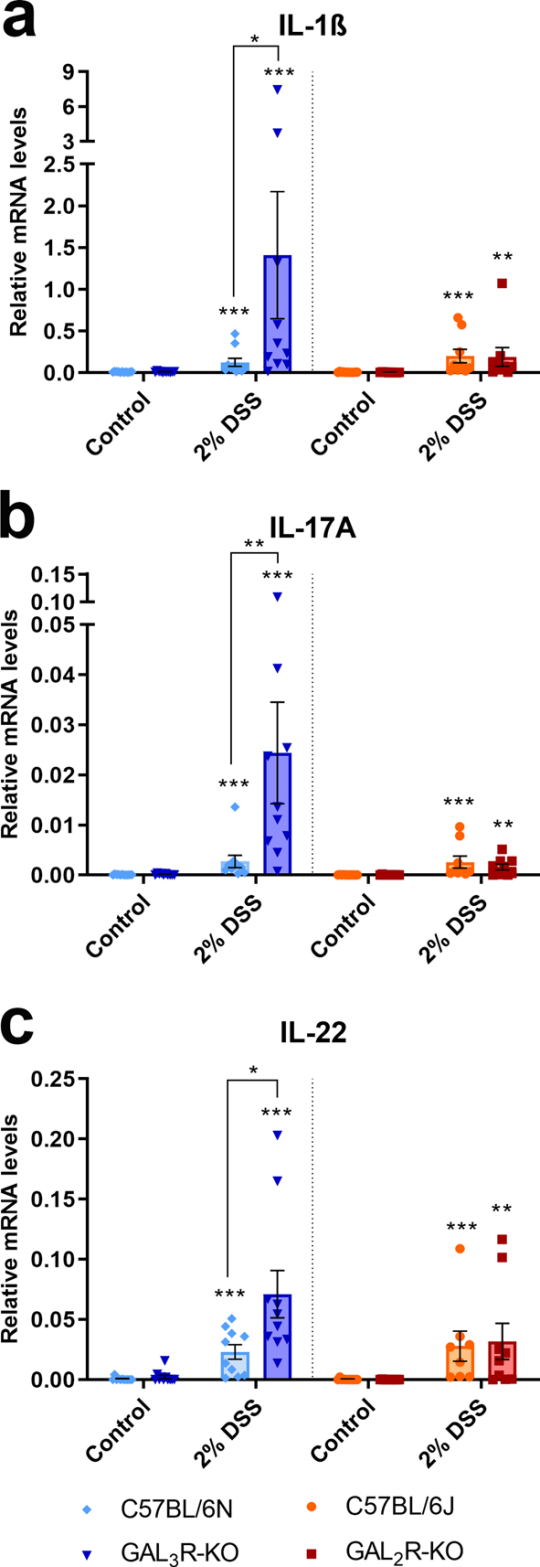
Relative mRNA expression levels of IL-1β (**A**), IL-17A (**B**) and IL-22 (**C**) in the colon of GAL_3_R-KO, GAL_2_R-KO and corresponding WT mice. Levels were determined relative to the housekeeping gene HPRT. Data represent means ± SEM. n = 7–11. Data were analyzed by Kruskal–Wallis test followed by Mann–Whitney U test. *p < 0.05; **p < 0.01; ***p < 0.001 versus corresponding controls or as indicated.

Induction of colitis significantly elevated the mRNA and protein expression of the majority of analyzed cytokines and chemokines in all DSS-treated groups compared to corresponding control groups (*p* < 0.05) (Figs. 5, 6, S3). In agreement with the increase in MPO levels and numbers of infiltrating neutrophils, transcript levels of the neutrophil-attracting chemokines CXCL1 and CCL2 were up to 5.4-fold higher in DSS-treated GAL_3_R-KOs compared to GAL_3_R-WT mice (CXCL1, *p* = 0.003; CCL2, *p* = 0.038) (Figs. 5a,c). Consistent with mRNA levels, the protein levels of these chemokines were up to threefold higher in DSS-treated GAL_3_R-KO compared to GAL_3_R-WT mice (CXCL1, *p* = 0.049; CCL2, *p* = 0.003) (Fig. 5b,d). The cytokines IL-6 and IFNγ showed a trend toward higher mRNA expression in DSS-treated GAL_3_R-KOs compared to treated GAL_3_R-WT animals (IL-6, *p* = 0.076; IFNγ, *p* = 0.059) (Fig. 5e,g). In agreement, plasma concentrations of these cytokines were significantly higher in DSS-treated GAL_3_R-KOs compared to GAL_3_R-WT mice (IL-6, *p* = 0.011; IFNγ, *p* = 0.004) (Fig. 5f,h). IFNγ concentration was 21-fold higher in DSS-treated GAL_3_R-KOs compared to treated GAL_3_R-WT animals (Fig. 5h). TNFα mRNA also showed a trend toward higher mRNA expression in DSS-treated GAL_3_R-KOs compared to treated GAL_3_R-WT animals (*p* = 0.070), but TNFα was not detected in plasma of GAL_3_R-KO and GAL_3_R-WT animals (Fig. 5i,j). In addition, mRNA expression of the predominantly pro-inflammatory cytokines IL-1β, IL-17A and IL-22 was up to 11.3-fold higher in DSS-treated GAL_3_R-KO compared to GAL_3_R-WT mice (IL-1β, *p* = 0.017; IL-17A, *p* = 0.005; IL-22, *p* = 0.019) (Fig. 6). Interestingly, upon colitis induction, IL-5 mRNA was significantly reduced only in DSS-treated GAL_3_R-WT mice compared to corresponding controls (*p* = 0.034) but was not influenced by DSS in any other group. DSS-treated GAL_3_R-KOs exhibited significantly higher IL-5 mRNA levels compared to GAL_3_R-WT mice (*p* = 0.009), albeit the levels were similar between healthy and treated GAL_3_R-KO animals (Fig. S3a). Relative mRNA expression levels of IL-10, IL-23 and TGFβ were not affected by DSS in any treated group (Fig. S3b-d). Transcript and protein levels of all analyzed cytokines and chemokines were unaffected by GAL_2_R loss independent of treatment (Figs. 5, 6, S3).

### The intestinal microbiota in DSS-induced colitis is influenced by GAL_3_R deletion

**Figure 7:**
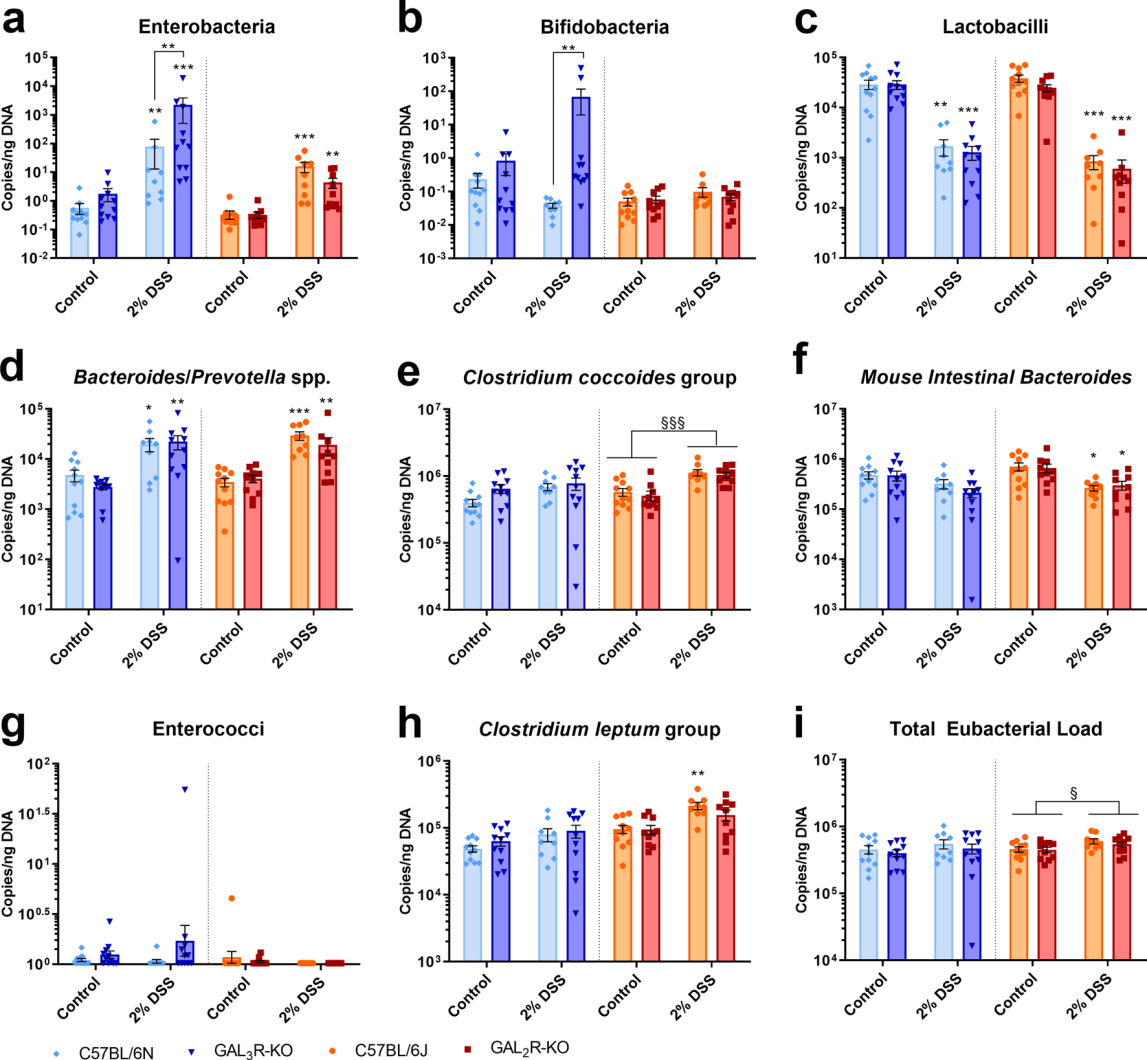
Changes of intestinal microbiota in GAL_3_R-KO, GAL_2_R-KO and corresponding WT mice following colitis induction with 2% DSS. Cecal contents were analyzed for main commensal bacterial groups, including enterobacteria (**a**), bifidobacteria (**b**), lactobacilli (**c**), *Bacteroides/Prevotella* spp. (**d**), members of the *Clostridium coccoides* group (**e**), *Mouse Intestinal Bacteroides* (**f**), enterococci (**g**), members of the *Clostridium leptum* group (H) and the total eubacterial load (**i**). Data represent means ± SEM. n = 9–11. Data were analyzed by two-way ANOVA, followed by Tukey’s test (main effect treatment: ^§^*p* < 0.05; ^§§§^*p* < 0.001) or by Kruskal–Wallis test followed by Mann–Whitney U test. *p < 0.5; **p < 0.01; ***p < 0.001 versus corresponding controls or as indicated.

The overall composition of the gut microbiota of healthy mice remained unaffected by loss of GAL_2_R and GAL_3_R (Fig. 7), which indicates that strain-specific susceptibility to DSS-induced colitis is independent of the microbiota.

Colitis induction resulted in enterobacterial enrichment in the cecal contents of all treated groups, independent of genotype (*p* < 0.05). Importantly, in DSS-treated GAL_3_R-KOs, enterobacterial copy numbers were 31.8-fold higher compared to DSS-treated GAL_3_R-WT animals (*p* = 0.009) (Fig. 7a). Remarkably, although copy numbers of bifidobacteria were similar in the cecal contents of DSS-treated mice compared to corresponding healthy controls (*p* < 0.05), gene numbers of bifidobacteria were 460.3-fold higher in DSS-treated GAL_3_R-KOs compared to DSS-treated GAL_3_R-WT mice (*p* = 0.001) (Fig. 7b). Following colitis induction copy numbers of lactobacilli were decreased and numbers of *Bacteroides/Prevotella spp.* were increased in GAL_3_R-KO and GAL_3_R-WT mice (*p* < 0.05), with no differences between genotypes (Fig. 7c,d). Other bacterial taxa as well as the total eubacterial load remained unaffected by treatment or genotype in GAL_3_R-KO and GAL_3_R-WT mice (Fig. 7e–i).

Following colitis induction, GAL_2_R-KO and GAL_2_R-WT mice exhibited similar changes to the gut microbiome as observed in GAL_3_R-KO and GAL_3_R-WT mice; however, gene numbers were similar between DSS-treated GAL_2_R-KO and GAL_2_R-WT mice (Fig. 7). In addition, DSS treatment altered the copy numbers of members of the *Clostridium coccoides* and *Clostridium leptum* group, the *Mouse Intestinal Bacteroides*, and the total eubacterial load in GAL_2_R-KOs and GAL_2_R-WT mice, independent of genotype (Fig. 7e,f,h,i). However, these differences in copy numbers between untreated and treated groups were smaller than one order of magnitude, and because of inter-assay variations these changes lack biological relevance.

### Expression profiles of the galanin system are not affected by GAL_2_R or GAL_3_R deletion

Since our findings could be influenced by compensatory regulation of other members of the galanin system in KO animals, we analyzed mRNA expression levels of galanin and GALRs in colon tissue of experimental animals. Loss of GAL_2_R or GAL_3_R did not change the expression of the galanin system, independent of treatment (Fig. S4). Galanin mRNA levels remained unaffected by DSS, independent of genotype (Fig. S4a). In all healthy WT mice, we measured similar levels of GAL_1_R and GAL_2_R mRNA, whereas GAL_3_R mRNA expression was low (Fig. S4b–d). Colitis induction resulted in significant downregulation of GAL_1_R mRNA in GAL_3_R-KO and GAL_3_R-WT mice (main treatment effect, *p* = 0.048), but not in GAL_2_R-KO and GAL_2_R-WT mice (Fig. S4b). Expression levels of GAL_2_R and GAL_3_R were not affected by DSS (Fig. S4c and d).

## Discussion

In this study, we found expression of GAL_2_R and GAL_3_R, but not GAL_1_R, on granulocytes in the colon of IBD patients. Remarkably, the presence of GAL_2_R and GAL_3_R was associated with higher disease activity. In a murine colitis model, we observed exacerbated histological damage, amplified inflammatory response and considerable alterations of the gut microbiome if GAL_3_R, but not GAL_2_R, was lacking.

In the literature, involvement of the galanin system in immunity and inflammation is well established. Some studies also indicated a role in colitis^22,27,29,30^. However, to date, data on protein expression of GAL_2_R or GAL_3_R in the colon are missing and the GALR subtype(s) mediating galanin’s effects on colitis remain(s) to be elucidated. As galanin expression is increased in the inflamed GIT^21,22^, we would have expected alterations in the colonic expression of GALRs during colitis. However, in the present study IHC analysis revealed no GALR protein expression by the colonic mucosa, neither in healthy individuals, nor in IBD patients. In contrast to our findings, strongly increased GAL_1_R expression under inflammatory conditions has previously been reported in human colonic cell lines and in human and mouse colon tissue^27,31^. However, another study found no difference in the density of galanin-specific binding sites in colon tissue of healthy individuals and IBD patients^24^. Interestingly, we observed protein expression of GAL_2_R and GAL_3_R, but not GAL_1_R, on granulocytes in colonic mucosa of IBD patients. This could explain why lack of GAL_1_R had no influence on murine colitis^33^. Although only a subset of granulocytes in the colon of IBD patients expressed GAL_2_R or GAL_3_R, these cells could potentially participate in IBD-related inflammatory processes by influencing other neighboring immune cells. However, it is not clear which granulocytes express GALRs, as this specific subgroup needs to be defined by the co-expression of other markers. In agreement, we found only a subset of macrophages expresses GAL_1_R and GAL_2_R in a xanthelasma of the skin^10^, a small proportion of granulocytes in human glioma and pituitary adenoma expresses GAL_2_R, and subpopulations of glioma-associated macrophages/microglia express GAL_1_R, GAL_2_R and GAL_3_R, with GAL_3_R being the most abundant GALR subtype in tumor-infiltrating immune cells^38^. These findings indicate that GAL_2_R and/or GAL_3_R signaling on granulocytes in the colonic mucosa could contribute to IBD progression.

Indeed, the results of the present study partially confirm this hypothesis as we found that histologic damage and immune activation associated with DSS-induced colitis are exaggerated in GAL_3_R-KO, but not in GAL_2_R-KO mice. Due to dissimilar genetic backgrounds of the GALR-KO mouse strains, we cannot exclude that the results might be different if the strains were on the same background; however, since only a few disease-related parameters were just marginally different between GAL_2_R-WT (C57BL/6J) and GAL_3_R-WT (C57BL/6N) mice (data not shown), there is no reason to suspect this.

Loss of GAL_3_R aggravates colonic inflammation following DSS treatment, however, it does not influence disease-related parameters including colon weight, colon length or disease activity score. Nevertheless, this indicates that activation of GAL_3_R signaling could improve disease outcome. Support for this is given by a study showing beneficial effects of galanin treatment on TNBS-induced colitis in rats. Interestingly, galanin was more effective in acute compared to chronic colitis^29,30^. In contrast, blocking GALR signaling with the non-selective GALR antagonist M35 in mice did not affect the body weight loss in DSS-induced colitis but, compared to vehicle-treated mice, caused a more rapid recovery of the body weight after discontinuation of DSS treatment^22^. However, it is unclear if M35 targets GAL_3_R at all^39^. Possible explanations for the discrepant effects of pharmacological activation and blocking of GALRs are the use of different animal species, as well as different colitis models. The TNBS-induced colitis model is preferentially used to study the pathophysiology related to CD, whereas DSS-induced colitis more closely resembles UC^40,41^. Nevertheless, Yamaguchi et al. showed that mucosal-type mast cells (MMCs) express GAL_3_R mRNA at 30-fold higher levels compared to GAL_2_R mRNA^22^. Furthermore, intraperitoneal application of M35 to DSS-treated mice diminished MMC numbers in inflamed colon^22^. As mast cells also play an important role in animal models of colitis and in IBD in humans^42–44^, these results support the involvement of GAL_3_R in murine experimental colitis.

In agreement with its putative role in IBD, we reported anti-inflammatory effects of GAL_3_R signaling on neutrophil-related MPO levels in murine arthritis^13^. In contrast, pro-inflammatory properties of GAL_3_R signaling were observed in murine psoriasis^14^ and pancreatitis^15^. Although such biphasic effects are well known for regulatory peptide systems, it is still unclear how such functions are exerted. Accumulating data indicate that the microenvironment as well as the activation state of immune cells influences the mode of action of regulatory peptides and their receptors. For example, we observed that galanin treatment enhanced IL-12/18-stimulated IFNγ secretion by NK cells when they were seeded at a high confluency. When the confluency was low, exogenous galanin reduced the amount of IFNγ secreted^9^. Furthermore, galanin treatment can increase or decrease cytokine/chemokine expression levels of macrophages depending on their differentiation and polarization status^10^. Consequently, it is not surprising that GAL_3_R signaling can have pro- and anti-inflammatory effects in diseases which differ in their organ localization and immune cell profiles. Regarding colitis, it can be speculated that GAL_3_R participates in inflammatory processes taking place in the colon and/or directly influences immune cell functions. Remarkably, GAL_3_R deletion affected mRNA and protein levels of inflammatory cytokines and chemokines during colitis. The majority of these cytokines and chemokines were previously implicated in IBD pathology^45–48^. Interestingly, GAL_3_R knockout was able to modulate the expression of members of the type-1, type-2 and IL-1 family of cytokines, as well as expression of neutrophil-attracting chemokines. Likewise, in psoriatric skin, lack of GAL_3_R altered the cytokine expression profile^14^. As some of these cytokines and chemokines impact other immune cell types besides neutrophils (e.g. macrophages or T cells), and as these cell types are involved in IBD^46^, the number of these immune cells could also be altered in colon tissue of GAL_3_R-KO mice. However, we observed that in psoriatric skin only the numbers of neutrophils but not the numbers of macrophages or mast cells were affected by loss of the GAL_3_R^14^.

In psoriasis, we found GAL_3_R to be expressed by dermal blood vessels and to influence neovascularization^14^. Angiogenesis is also an important pathogenic factor in IBD progression^49^. Importantly, as we also observed GAL_3_R-positive staining on blood vessels in the human colon, GAL_3_R might also be involved in neovascularization during IBD.

Current research is concentrating on the role of the gut microbiota in IBD pathophysiology. It has become apparent that the intestinal microbiota not only influences disease susceptibility^35,36^, but also affects inflammatory processes in general^50,51^. Several studies have shown direct involvement of neuropeptide systems in maintaining microbiome homeostasis^52,53^. The composition of commensal bacteria was unaffected by loss of GAL_3_R in healthy mice; therefore, increased disease susceptibility due to alterations to the microbiota in GAL_3_R-KO mice could be excluded. Colitis induction with DSS resulted in increased abundances of enterobacteria and *Bacteroides/Prevotella* spp. and reductions of lactobacilli in the cecum. These changes are frequently seen in inflammatory conditions affecting the GIT^54,55^. Surprisingly, in DSS-treated GAL_3_R-KO mice, enterobacterial enrichment was more pronounced and the copy numbers of bifidobacteria were elevated. It is still controversial, however, whether this altered balance of gut microbiota constituents in DSS-treated GAL_3_R-KOs is a cause or consequence of the intestinal inflammation^35^. On the one hand, the inflammatory environment in the gut seems to offer a growth advantage to Enterobacteriaceae, resulting in the enterobacterial enrichment observed in colitis. On the other hand, Enterobacteriaceae were shown to boost inflammation and to contribute to disease development^56,57^. In contrast, Bifidobacteriaceae have potential anti-inflammatory properties, as they alleviated experimental colitis severity^58^ and were reduced in the microbiome of UC patients^59^. The simultaneous overgrowth of potential pro- and anti-inflammatory bacterial taxa in the cecum of DSS-treated GAL_3_R-KOs might appear contradictory but it could be acting as a feedback loop, which is well known in inflammatory processes. In general, these data clearly support the initial hypothesis that during colitis the gut microbiota is altered in GAL_3_R-KO mice.

In conclusion, the present study strongly supports the involvement of GAL_3_R in IBD pathophysiology. In experimental colitis, histologic damage and immune activation were aggravated and changes to the microbiota more pronounced in the absence of GAL_3_R. Thus, this study identifies activation of GAL_3_R signaling as a possible target for new treatment strategies to combat IBD. Future studies are, however, hampered by the current lack of specific and selective GAL_3_R agonists^5^. Nevertheless, the need for further research to elucidate the role of GAL_3_R in IBD in more detail is clearly indicated.

## Materials and methods

### Human patients and tissue samples

The study was approved by the local ethics committee of the Land Salzburg, Austria, (415-E/2080/5-2016) and conducted in accordance with the Helsinki Declaration of 1975 (revised 2013).

The participants were recruited at the Gastroenterological Divisions of Pediatric and Internal Clinics at the University Hospital in Salzburg, Austria. Patients with chronic abdominal pain, elevated fecal calprotectin or signs and symptoms of IBD underwent diagnostic colonoscopy, where biopsy specimens were taken from the ascending colon. Pathologists evaluated crypt architecture, acute and chronic inflammation, and regeneration of the epithelium using an arbitrary score from 0 to 3. Patients with proven IBD were assigned to our study group [CD (n = 10; 20% females; mean age 20.1 ± 5.4 years) or UC (n = 5; 60% females; mean age 20.2 ± 4.2 years)]. Healthy controls (n = 9; 77% females; mean age 18.7 ± 3.9 years) were selected to the study group when they had no inflammation or alterations in crypt architecture, or other abnormalities on endoscopic and histomorphologic evaluation. The latter was verified by a pathologist. Reasons for performing an endoscopy in healthy subjects were: hematochezia (n = 1), diarrhea (n = 1), abdominal pain (n = 3), exclusion of IBD (n = 3), or constipation (n = 1). All participants signed written informed consent or informed consent was obtained from a parent and/or legal guardian if study participants were under the age of 18.

Colon biopsies were provided as formalin-fixed and paraffin-embedded (FFPE) tissue by the Institute of Pathology of the University Hospital Salzburg, Austria. Detailed information on patients is found in Supplementary Table S1.

### Experimental animals and induction of colitis

All animal procedures were approved by the ethical committee at the Federal Ministry of Science and Research of the Republic of Austria (GZ 66.010/0037-II/3b/2013) and conducted according to the Directive of the European Parliament and of the Council of 22 September 2010 (2010/63/EU). This study additionally adheres to standards articulated in the ARRIVE guidelines^60^.

In vivo experiments were conducted at the Medical University of Graz, Austria using 8–12-week old male GAL_2_R-KO (C57BL/6J background)^61^, GAL_3_R-KO (C57BL/6N background)^62^ and corresponding WT mice. Detailed information on animals and genotyping can be found in Supplementary Methods.

Mice were housed in groups of 2 or 3 animals per cage (one mouse in the GAL_2_R-KO/DSS group had to be single-housed due to aggressiveness) under controlled conditions (temperature 21 °C, humidity 50%) at a 12 h light/dark cycle (lights on/off at 0600/1800 h) in open-ventilated cages with wood chip bedding and a triangular wood pulp house as enrichment.

To induce acute colitis, mice (n = 9–11 per group) were treated with 2% DSS (36–50 kDa; MP Biochemicals, Illkirch, France), added to the drinking water, for 7 days ad libitum. Control animals received plain drinking water^55,63,64^. Animals were fed with standard rodent chow ad libitum.

Body weight, food and water intake were assessed daily at the same time of day (0900 h). Food and water intake were measured per cage and then divided by the number of mice in each cage to determine the daily intake per animal.

Detailed information on sample collection, assessment of the DAS^64,65^, evaluation of intestinal inflammation^66^, measurement of colonic MPO content^63^, real-time quantitative PCR (qPCR) analysis of cytokines/chemokines in colon tissue, and measurement of plasma cytokine/chemokine levels^64^ is given in Supplementary Methods.

### Immunohistochemistry

For IHC studies, human colon sections were stained with hGAL_1_R (GTX108207, 1:400; Genetex, Irvine, CA, USA), hGAL_2_R (customized: S4510-1, 1:400; PTG, Manchester, UK), and hGAL_3_R (GTX108163, 1:500; Genetex) as published recently^34^. Mouse colon sections were stained with mNIMP-R14 (ab2557, 1:100, Abcam, Cambridge, UK) as published previously^14^. Detailed information on the IHC protocol and quantification of IHC staining can be found in Supplementary Methods.

### Molecular analysis of microbiota in cecal contents

DNA was extracted from the contents of mouse cecum as described previously^53^. Total DNA was then quantified by using Quant-iT PicoGreen reagent (Thermo Fisher, Dreieich, Germany) and adjusted to 1 ng/µl. Bacterial groups abundant in the intestinal microbiota were assessed by qPCR with species-, genera-, or group-specific 16S rRNA gene primers (Tib MolBiol, Berlin, Germany) as described previously^53^. Gene copy numbers per nanogram DNA were determined.

### Statistical analysis

Statistical analysis was performed using Graph Pad Prism 8.0 (GraphPad Software Inc., San Diego, CA, USA) and SPSS 24.0 (IBM, Armonk, NY, USA). All data sets were tested for normal distribution using the Shapiro–Wilk test and for homogeneity of variances using the Levene test. Data on daily body weight, food and water intake were analyzed by two-way repeated measures (RM) ANOVA. Post hoc testing was performed with one-way RM ANOVA and Sidak’s multiple comparison test or with two-way ANOVA and Tukey’s test, as appropriate. Data consisting of one variable and two factors were analyzed by two-way ANOVA and Tukey’s test. If ANOVA assumptions were not met or if data sets were ordinal variables (semiquantitative scores), the non-parametric Kruskal–Wallis test followed by the Mann–Whitney U test was used. *p* values < 0.05 were regarded as statistically significant.

## Supplementary Information

Supplementary Information.

## Data Availability

The raw data supporting the conclusions of this article will be made available by the corresponding author, without undue reservation, to any qualified researcher.
