# Supplementary material for: Galanin receptor 3 attenuates inflammation and influences the gut microbiota in an experimental murine colitis model
Source: Sci Rep. 2021 Jan 12;11:564. doi: 10.1038/s41598-020-79456-y (PMC7803768; doi:10.1038/s41598-020-79456-y)
Supplement: Supplementary file 1 — Supplementary Information. [file 41598_2020_79456_MOESM1_ESM.pdf]

Supplementary Information for

Galanin receptor 3 attenuates inflammation and influences the gut microbiota in an experimental murine colitis model.

Susanne M Brunner[1,*], Florian Reichmann[2], Julia Leitner[1], Soraya Wölfl[3], Stefan Bereswill[4], Aitak Farzi[2], Anna-Maria Schneider[5], Eckhard Klieser[6], Daniel Neureiter[6], Michael Emberger[3], Markus M. Heimesaat[4], Daniel Weghuber[5], Roland Lang[7], Peter Holzer[2], Barbara Kofler[1]

[1]Research Program for Receptor Biochemistry and Tumor Metabolism, Department of Pediatrics, University Hospital of the Paracelsus Medical University, Muellner Hauptstr. 48, 5020 Salzburg, Austria.

[2]Research Unit of Translational Neurogastroenterology, Division of Pharmacology, Otto Loewi Research Center, Medical University of Graz, Universitätsplatz 4, 8010 Graz, Austria.

[3]Laboratory for Pathology Weger, Emberger, Strubergasse 20, 5020 Salzburg, Austria.

[4]Institute of Microbiology, Infectious Diseases and Immunology, Charité – University Medicine Berlin, Garystr. 5, 14195 Berlin, Germany.

[5]Department of Pediatrics, University Hospital of the Paracelsus Medical University, Muellner Hauptstr. 48, 5020 Salzburg, Austria.

[6]Institute of Pathology, University Hospital of the Paracelsus Medical University, Muellner Hauptstr. 48, 5020 Salzburg, Austria.

[7]Department of Dermatology and Allergology, University Hospital of the Paracelsus Medical University, Muellner Hauptstr. 48, 5020 Salzburg, Austria.

*Corresponding author:
Susanne Brunner
Email: su.brunner@salk.at

Supplementary methods

Experimental animals

Mice with an early termination mutation in the intron of the GAL_2R gene on the C57BL/6J background were kindly provided by Marina Picciotto's lab (Yale University, New Haven, CT, USA) [1]. GAL_3R-KO mice were initially obtained from the European Mouse Mutant Archive (EMMA). They were generated by homologous recombination targeting both coding exons. The mouse line was backcrossed to the C57BL/6N lineage for at least 7 additional generations. The mouse line underwent a thorough phenotypical characterization in our lab [2].
WT and KO animals used in this study were bred at the animal facility of the Paracelsus Medical University, Salzburg, Austria, and originated from homozygous breeders. To avoid genetic drift, all genotypes were crossed back twice to the C57BL/6N or J background (Charles River, Germany) every 8-10 generations. All animals were genotyped before being used in experiments as described previously [2, 3]. At the age of about 6 weeks, experimental animals were transferred to the animal facility of the Medical University of Graz, Austria, where the experiments were conducted. Before the start of the experiments, the mice were allowed to habituate to the animal facility for at least one week.

Evaluation of disease-related parameters and sample collection

On treatment day 7, a disease activity score (DAS) was calculated, which combined the scores of three parameters: weight difference between start and end of DSS treatment (0 - weight increase ≥ 1 g, 1 - weight increase < 1 g, 2 - weight decrease < 1 g, 3 - weight decrease ≥ 1 g), stool consistency (0 - normal stool, 1 - soft but formed stool, 2 - loose stool), and presence of blood in feces and/or the perianal region (0 - no trace of blood, 1 - positive hemoccult test, 2 - trace of blood or bloody perianal region) [4, 5]. Presence of blood in feces was determined with the hemoccult test (Beckman Coulter, Krefeld, Germany).
At the end of the treatment period, unfasted mice were deeply anesthetized with pentobarbital (150 mg/kg i.p.) and blood was collected by cardiac puncture in a syringe containing 100 µl sodium citrate (3.8% w/v) as anticoagulant. Blood was centrifuged at 2,500 g for 10 min at 4°C. Plasma was collected and stored at -70°C until analysis. Mice were euthanized by cervical dislocation and the large intestines, including cecum, colon, and rectum, were dissected and colon length (from end of the cecum to the rectum) was measured. Subsequently, the colon was flushed with ice-cold phosphate-buffered saline (PBS), tapped dry with a paper towel and weighed. The colon was cut into pieces. The most distal part of the colon was fixed in 4% paraformaldehyde for a maximum of 3.5 hours, washed with PBS and then embedded into paraffin (FFPE). Additional pieces were snap-frozen in liquid nitrogen and stored at -70°C. The contents of the cecum were stored at -70°C. The spleen was excised and weighed.

Evaluation of intestinal inflammation

Longitudinal sections (5 µm) of FFPE colon tissue were cut and stained with hematoxylin and eosin (HE). Digital micrographs were taken with a Moticam 5+ camera using Motic Images Plus 2.0 software (Motic, Wetzlar, Germany). Inflammatory alterations in the colon tissue were evaluated independently by a researcher and a pathologist blinded to information on treatment or genotype. Semiquantitative scoring was performed according to Erben et al. [6] and is described in detail in Supplementary Table S2.

Colonic myeloperoxidase content

The tissue levels of MPO were used as an index of inflammation-associated infiltration by neutrophils into the colon tissue. Murine colon samples were homogenized and MPO levels were measured by ELISA (Hycult Biotechnology, Uden, The Netherlands) [7].

Real-time quantitative PCR (qPCR)

RNA was extracted from colon samples using the RNeasy Mini Kit (Qiagen, Hilden, Austria). Two micrograms of RNA were submitted to a reverse transcription reaction using random hexamer primers and Maxima reverse transcriptase (Thermo Fisher, Vienna, Austria). In colon, mRNA expression levels of the various members of the galanin system [ppGAL, GAL_1R, GAL_2R, and GAL_3R], inflammatory cytokines [interleukin (IL) 1β, IL-5, IL-6, IL-10, IL-17A, IL-22 and IL-23, interferon γ (IFNγ), transforming growth factor β (TGFβ) and tumor necrosis factor α (TNFα) and chemokines [chemokine (C-X-C motif) ligand 1 (CXCL1) and chemokine (C-C motif) ligand 2 (CCL2)] were measured in duplicates by qPCR

using the SYBR Green iQ Supermix (Bio-Rad, Vienna, Austria) on a qTower 2.0 system (Analytik Jena, Vienna, Austria). Hypoxanthine-guanine phosphoribosyltransferase (HPRT) served as a housekeeping gene. Specific primer sequences are shown in Supplementary Table S3 and cycling conditions are listed in Supplementary Table S4. Quantification cycles (Cq) >32.0 were rated as negative expression. Relative mRNA expression levels of target genes were calculated relative to expression of HPRT by the ΔΔCq method [$2^{-(\text{Cq of gene of interest-Cq of HPRT})}$].

Plasma cytokine levels

Concentrations of IL-1β, IL-6, IL-10, IL-21, TNFα, IFNγ, CXCL1 and CCL2 were quantified in plasma in duplicates by using the ProcartaPlex™ Multiplex immunoassay (eBioscience, Thermo Fisher, Vienna, Austria) on a Bio-Plex 200 system with Bio-Plex 5.0 Software (Bio-Rad, Hercules, CA, USA). Values below the detection limit were set to zero [5].

Immunohistochemistry

For IHC studies, human colon sections were stained with $hGAL_1R$ (GTX108207, 1:400; Genetex, Irvine, CA, USA), $hGAL_2R$ (customized: S4510-1, 1:400; PTG, Manchester, UK), and $hGAL_3R$ (GTX108163, 1:500; Genetex, Irvine, CA, USA). Antibodies against GALRs were extensively validated, recently [8]. Sections from mouse colon were stained with mNIMP-R14 (ab2557, 1:100, Abcam, Cambridge, UK) as published previously [9]. Primary antibodies were diluted in Dako Antibody Diluent with Background Reducing Components (DAKO, Glostrup, Denmark). FFPE human and mouse colon tissue was sectioned to 4-5 µm, mounted onto SuperFrost Plus Microscope glass slides (Thermo Fisher) and dried at 60°C for 1 h. After deparaffinization and rehydration, heat-induced epitope retrieval was performed with 10 mM Tris-HCl, pH 8, 1 mM EDTA, 0.05% Tween-20 for 40 min at 95°C for human tissue or with 10 mM Tris-HCl, pH 9, 1 mM EDTA at 60°C overnight for murine tissue. Immunohistochemical staining was performed using the Envision+ System-HRP (DAB) Kit (DAKO) according to the manufacturer's instructions, including blocking of endogenous peroxidases. Slides were incubated with primary antibody for 60 min at room temperature (RT) or 40 min at 37°C. After washing with 1x PBS containing 0.5% Tween-20, human tissue was incubated with the Envision+ HRP-labeled polymer (anti-rabbit, second antibody, ready-to-use, DAKO) and murine tissue with rabbit anti-rat immunoglobulins/HRP (P0450, 1:50, Dako) for 30 min at RT. Another washing step was followed by visualization with Envision+ Liquid DAB+ Chromogen (diaminobenzidine solution) for 10 min at RT. Slides were washed in tap water and counterstained in Mayer's hemalum solution (Merck KGaA, Darmstadt, Germany) for 3-5 min. Slides were briefly rinsed in 3% hydrochloric acid in ethanol and rinsed for 10 min under running tap water. After dehydration with 2-propanol, the slides were incubated in xylene and mounted in Histokitt (Karl Hecht GmbH & Co. KG, Sondheim, Germany). Digital micrographs were taken with a Moticam 5+ camera using Motic Images Plus 2.0 software (Motic, Wetzlar, Germany).

For each round of IHC staining (human colon only), appropriate control sections were included as quality control. The cell line SH-SY5Y transfected with either human GAL_1R or GAL_2R was used as a positive control for GAL_1R and GAL_2R staining, respectively [8]. Human skin sections were used as a positive control for GAL_3R staining (positive blood vessels) [9]. Furthermore, as a control, the primary antibody was omitted (human and murine colon).

Quantification of IHC staining in human sections was performed independently by a researcher and a pathologist blinded to information regarding the disease group. Per colon specimen the total number of neutrophilic granulocytes and the number of GAL_2R- and GAL_3R-positive neutrophils were counted in three high power fields (HPF). Neutrophils were determined by their characteristic neutrophil morphology.

Quantification of IHC staining in murine colon was performed independently by two researchers blinded to information on treatment and genotype. The number of infiltrated neutrophils was determined by counting NIMP-R14$^+$ cells in the colonic mucosa and submucosa. NIMP-R14$^+$ cells inside blood vessels were disregarded as well as positively stained cells lacking characteristic neutrophil morphology. Up to four sections per mouse were evaluated. Neutrophil numbers were normalized to the total evaluated area of colon mucosa and submucosa. The area was assessed in mm^2 using Image J software.

Supplementary figures

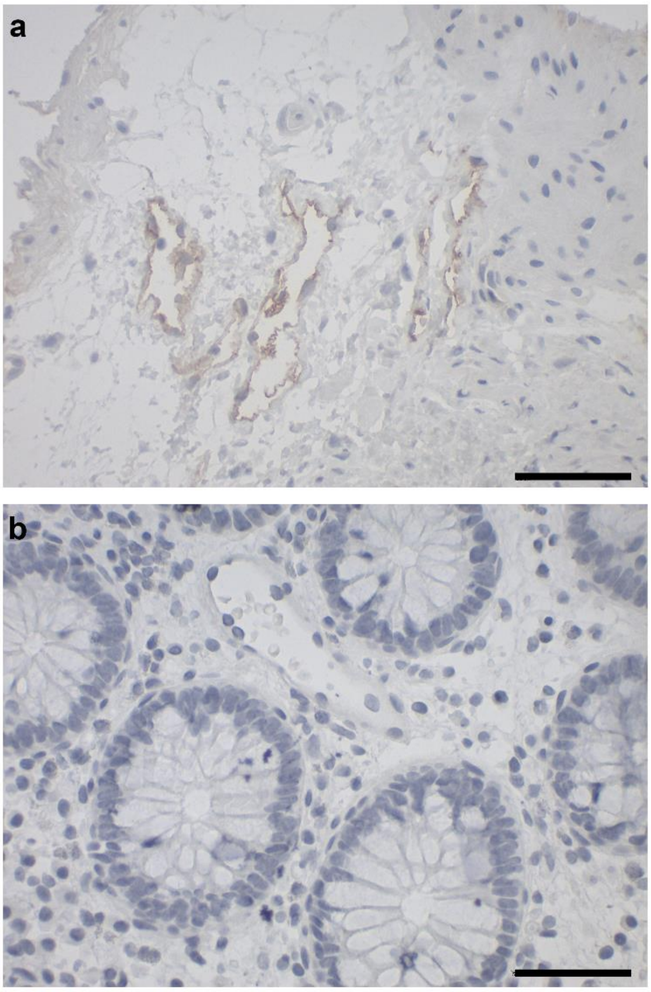

Figure S1. Representative images of IHC staining of GAL_3R in human colon (case 13, a) and of a negative control with second antibody only staining (b). Blood vessels are GAL_3R-positive (a). Scale bar: 50 µm.

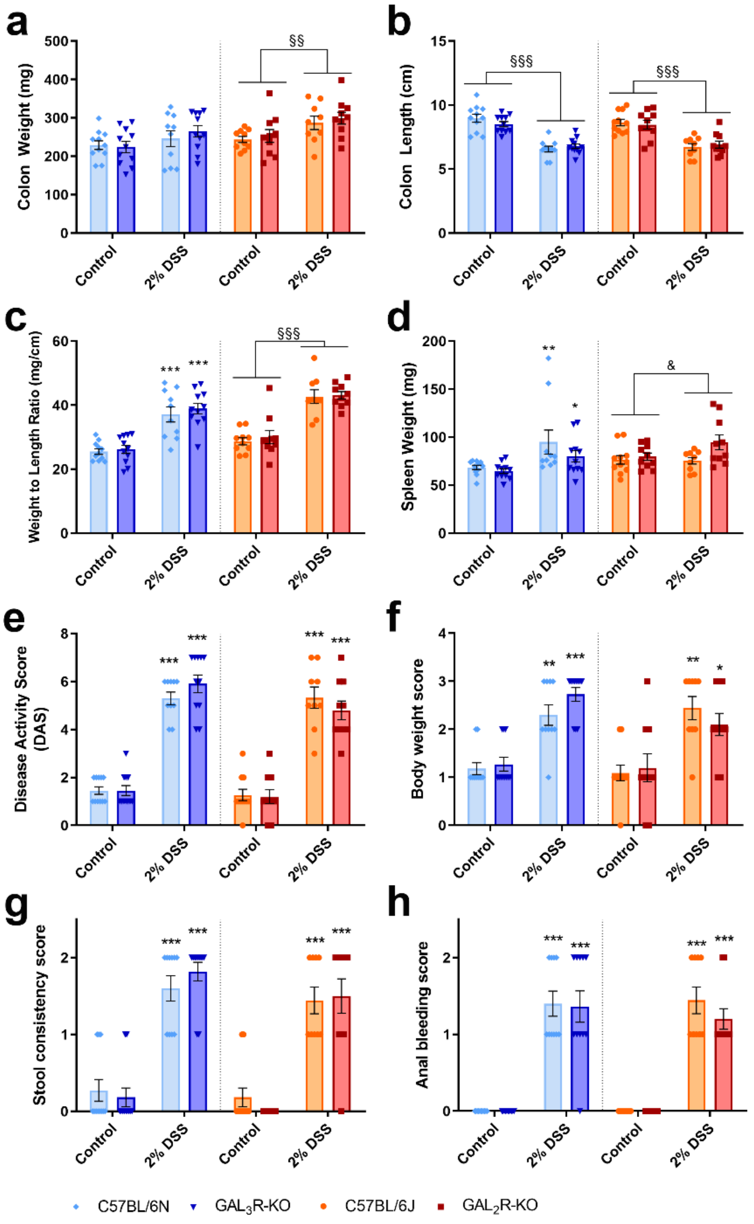

Figure S2. Disease-related parameters analyzed on day 7 of DSS treatment in GAL_3R-KO, GAL_2R-KO and corresponding WT mice. Colon weight (a), colon length (b), colon weight to length ratio (c) and spleen weight (d), cumulative disease activity score (DAS) (e) and DAS single scores (f-h). Data represent means ± SEM. n=9-11. Data were analyzed with two-way ANOVA, followed by Tukey's test (main effect treatment: §§§<p0.001; main effect genotype: &p<0.05), or by Kruskal-Wallis-Test followed by Mann-Whitney-U Test, as appropriate (*p<0.05, **p<0.01, ***p<0.001 vs. corresponding control group).

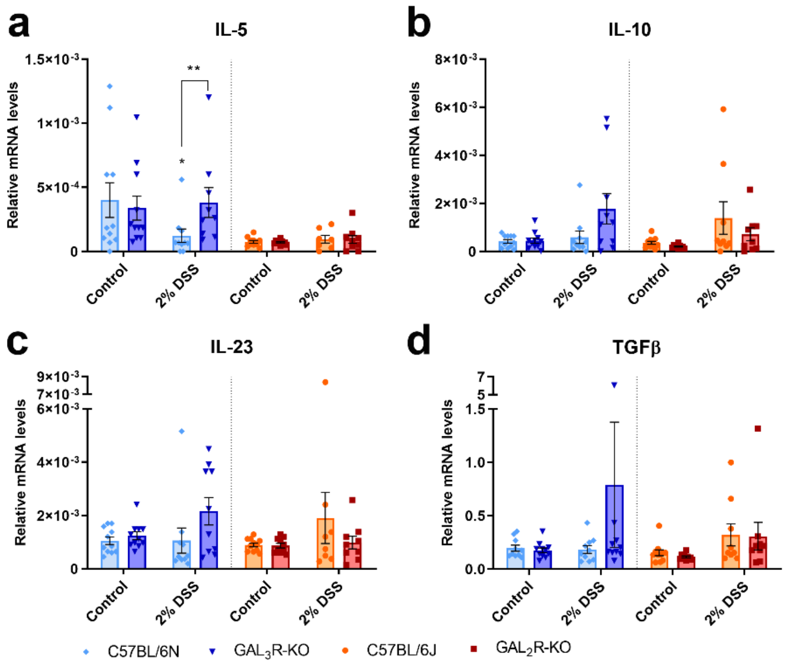

Figure S3. Relative mRNA expression levels of IL-5 (a), IL-10 (b), IL-23 (c) and TGFβ (d) in colon of GAL3R-KO, GAL2R-KO and corresponding WT mice determined relative to the housekeeping gene HPRT. Data represent means ± SEM. n=7-11. Data were analyzed by Kruskal-Wallis test followed by Mann-Whitney U test. *p<0.05, **p<0.01 vs. corresponding controls or as indicated.

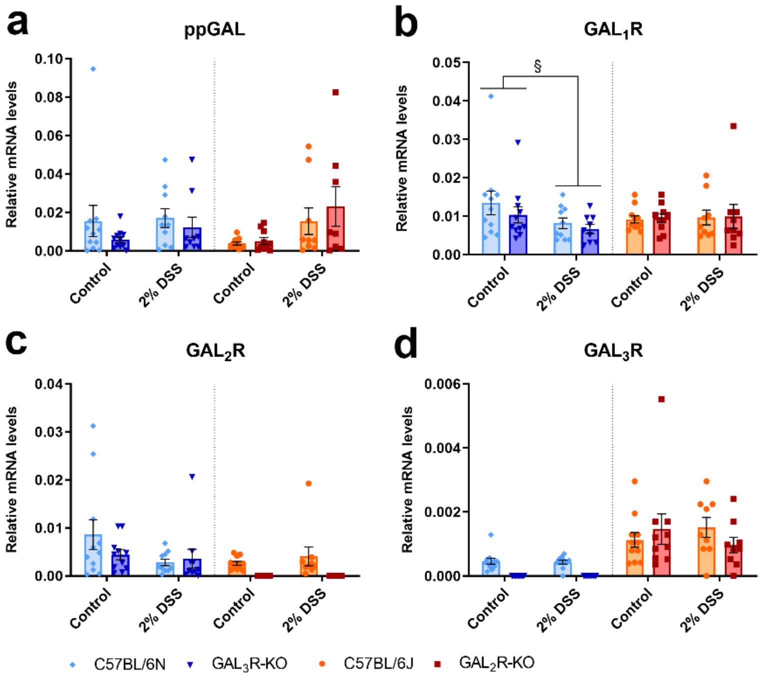

Figure S4. Relative mRNA expression levels of pre-pro-galanin (ppGAL, a), GAL_1-R (b), GAL_2-R (c) and GAL_3-R (d) in distal colon of GAL_3R-KO, GAL_2R-KO and corresponding WT mice determined relative to the housekeeping gene HPRT. Data represent means ± SEM. n=8-11. Data were analyzed with two-way ANOVA, followed by Tukey's test. Main effect treatment: §p<0.05.

Supplementary tables

Supplementary Table S1: Detailed information on human colon samples.

Case No.	Age (years)	Sex	Study group	Reason for endoscopy
**1**	16.6	m	Healthy	Exclusion of celiac disease
**2**	18.0	m	Healthy	Abdominal pain
**3**	20.9	f	Healthy	Diarrhea
**4**	24.6	f	Healthy	Exclusion of Crohn’s disease
**5**	18.7	f	Healthy	Chronic obstipation
**6**	22.5	f	Healthy	Exclusion of Crohn’s disease
**7**	10.1	f	Healthy	Abdominal pain
**8**	17.5	f	Healthy	Abdominal discomfort
**9**	19.7	f	Healthy	Hematochezia
**10**	10.9	m	Crohn’s disease	Diagnostic endoscopy
**11**	15.5	m	Crohn’s disease	Diagnostic endoscopy
**12**	18.0	f	Crohn’s disease	Diagnostic endoscopy
**13**	19.1	m	Crohn’s disease	Diagnostic endoscopy
**14**	24.8	m	Crohn’s disease	Diagnostic endoscopy
**15**	26.4	f	Crohn’s disease	Diagnostic endoscopy
**16**	26.7	m	Crohn’s disease	Diagnostic endoscopy
**17**	23.5	m	Crohn’s disease	Diagnostic endoscopy
**18**	12.6	m	Crohn’s disease	Diagnostic endoscopy
**19**	23.9	m	Crohn’s disease	Diagnostic endoscopy
**20**	13.8	f	Ulcerative colitis	Diagnostic endoscopy
**21**	17.7	m	Ulcerative colitis	Diagnostic endoscopy
**22**	20.4	f	Ulcerative colitis	Diagnostic endoscopy
**23**	25.5	f	Ulcerative colitis	Diagnostic endoscopy
**24**	23.6	m	Ulcerative colitis	Diagnostic endoscopy

Supplementary Table S2: Scoring scheme for DSS-induced colonic inflammation.

**INFLAMMATORY CELL INFILTRATE**		
**Severity**	**Extent**	**Score 1**
Minimal (<10%)		0
Mild (10-25% / scattered immune cells)	Mucosa	1
Moderate (26-50%)	Mucosa and submucosa	2
Marked (>51% / dense infiltration)	Transmural	3
**INTESTINAL ARCHITECTURE**		
**Epithelial changes**	**Mucosal architecture**	**Score 2**
Intact epithelium		0
Focal erosions		1
Erosions	± Focal ulcerations	2
	Extended ulcerations<br>± granulation tissue<br>± pseudopolyps	3
**TOTAL SCORE**		
**Sum of score 1 and 2**		**0-6**

Supplementary Table S3: Primer sequences for quantification of mRNA levels of inflammatory cytokines and chemokines and galanin system components in mouse colon.

Gene	Forward (5'-3')	Reverse (3'-5')
**CCL2**	AAGCCAGCTCTCTCTTCCTCCAC	TGAGTAGCAGCAGGTGAGTGGG
**CXCL1**	TGACCCTGAAGCTCCCTTGGTT	TCAGAAGCCAGCGTTCACCAGA
**ppGAL**	ACCAGGAAGTGTTGATGTGCC	TCTAGGTCTTCTGAGGAGGTGG
**GAL_1R**	CTGCCCTTACTGCTCATCTGC	TACAACGACCACCAGGACGG
**GAL_2R**	TCTCGCTGGACAGGTATCTGG	CTGACTGTAGTAGCTCAGGTAGGG
**GAL_3R**	GGCCGTCTCAGTGGATAGGT	AGCTTAGGTAGGGCGCGGA
**HPRT[1]**	GTCCCAGCGTCGTGATTAGC	GAGCAAGTCTTTCAGTCCTGTCC
**IL-1β**	AAAGACGGCACACCCACCCT	CCAGTTGGGGAACTCTGCAGAC
**IL-5**	ACGATGAGGCTTCCTGTCCCT	CATTTCCACAGTACCCCCACGG
**IL-6**	CCGGAGAGGAGACTTCACAGAGG	TCTGCAAGTGCATCATCGTTGT
**IL-10**	ACAGCCGGGAAGACAATAACT	TTCCGATAAGGCTTGGCAAC
**IL-17A**	AACCGTTCCACGTCACCCTG	GTCCAGCTTTCCCTCCGCAT
**IL-22**	AGGTGGTGCCTTTCCTGACCA	GCAGGTCCAGTTCCCCAATCG
**IL-23**	CCAGCGGGACATATGAATCTACTAA	TGCAAGCAGAACTGGCTGTTG
**IFNγ**	TAGCTCTGAGACAATGAACG	CACATCTATGCCACTTGAGT
**TGFβ**	ACCTTGGTAACCGGCTGCTGA	GCGCTGAATCGAAAGCCCTGT
**TNFα**	GGTCCCCAAAGGGATGAGAA	CTCAGCCACTCCAGCTGCTC

Legend:
[1]HPRT served as the housekeeping gene.

Supplementary Table S4: Cycling conditions used in quantitative real-time PCR analysis.

**Gene**	**Cycling conditions**
**Standard program**[1]	95°C, 3 min<br>(95°C, 15 sec; 64°C, 30 sec; 72°C 10 sec) 40x<br>95°C, 1 min<br>70°C, 1 min<br>Melt curve: 75>95°C, 5 sec per 0.5°C
**GAL_3R**	95°C, 3 min<br>(97°C, 15 sec; 63°C, 30 sec; 72°C 10 sec) 45x<br>95°C, 1 min<br>70°C, 1 min<br>Melt curve: 75>97°C, 5 sec per 0.5°C

Legend:
[1]The standard program is used for mRNA analysis of all gene targets listed in Supplementary table S3. Gene targets with deviating cycling programs are listed separately.

Supplementary Table S5: Detailed F statistics to statistical analyses of data sets on body weight, food intake and water intake in GAL_3R-KO, GAL_2R-KO and corresponding WT mice.

Dependent variable	Groups	Type of test	Subject effects	Main effects or Interactions	*df*	Error	F value	P value
**Body weight**	GAL_3R-KO/-WT	2-way RM[1] ANOVA	Within	D[2]	2.123	82.801	31.855	<0.001
**Body weight**	GAL_3R-KO/-WT	2-way RM ANOVA	Within	D*G[3]	2.123	82.801	4.622	0.011
**Body weight**	GAL_3R-KO/-WT	2-way RM ANOVA	Within	D*T[4]	2.123	82.801	37.987	<0.001
**Body weight**	GAL_3R-KO/-WT	2-way RM ANOVA	Within	D*G*T	2.123	82.801	4.733	0.010
**Body weight**	GAL_3R-KO/-WT	2-way ANOVA, day 4	-	G*T	1	39	4.518	0.040
**Body weight**	GAL_3R-KO/-WT	2-way ANOVA, day 5	-	G*T	1	39	5.384	0.026
**Body weight**	GAL_3R-KO/-WT	2-way ANOVA, day 6	-	G*T	1	39	7.938	0.008
**Body weight**	GAL_3R-KO/-WT	2-way ANOVA, day 7	-	G*T	1	39	9.072	0.005
**Body weight**	GAL_2R-KO/-WT	2-way RM ANOVA	Within	D	2.190	78.827	10.719	<0.001
**Body weight**	GAL_2R-KO/-WT	2-way RM ANOVA	Within	D*T	2.190	78.827	16.028	<0.001
**Body weight**	GAL_2R-KO/-WT	2-way RM ANOVA	Between	T	1	36	5.286	0.027
**Body weight**	GAL_2R-KO/-WT, controls	1-way RM ANOVA	Within	D	2.475	47.027	3.398	0.033
**Body weight**	GAL_2R-KO/-WT, DSS-treated	1-way RM ANOVA	Within	D	1.771	30.100	19.314	<0.001
**Food intake**	GAL_3R-KO/-WT	2-way RM ANOVA	Within	D	1.696	27.136	56.293	<0.001
**Food intake**	GAL_3R-KO/-WT	2-way RM ANOVA	Within	D*T	1.696	27.136	8.054	0.003
**Food intake**	GAL_3R-KO/-WT, controls	1-way RM ANOVA	Within	D	2.340	18.717	86.570	<0.001
**Food intake**	GAL_3R-KO/-WT, DSS-treated	1-way RM ANOVA	Within	D	1.464	11.710	21.058	<0.001
**Food intake**	GAL_2R-KO/-WT	2-way RM ANOVA	Within	D	2.678	37.493	10.256	<0.001
**Food intake**	GAL_2R-KO/-WT	2-way RM ANOVA	Within	D*T	2.678	37.493	7.280	0.001
**Food intake**	GAL_2R-KO/-WT	2-way RM ANOVA	Between	T	1	14	4.433	0.054
**Food intake**	GAL_2R-KO/-WT, DSS-treated	1-way RM ANOVA	Within	D	6	42	17.536	<0.001

Supplementary Table S5 *(continued)*

Dependent variable	Groups	Type of test	Subject effects	Main effects or Interactions	*df*	Error	F value	P value
**Water intake**	GAL_3R-KO/-WT	2-way RM ANOVA	Within	D	1.712	27.389	8.218	0.002
**Water intake**	GAL_3R-KO/-WT	2-way RM ANOVA	Within	D*T	1.712	27.389	10.628	0.001
**Water intake**	GAL_3R-KO/-WT, controls	1-way RM ANOVA	Within	D	6	48	8.443	<0.001
**Water intake**	GAL_3R-KO/-WT, DSS-treated	1-way RM ANOVA	Within	D	1.337	10.698	9.872	0.007
**Water intake**	GAL_2R-KO/-WT	2-way RM ANOVA	Within	D	2.039	28.540	30.256	<0.001
**Water intake**	GAL_2R-KO/-WT	2-way RM ANOVA	Within	D*T	2.039	28.540	6.695	0.004
**Water intake**	GAL_2R-KO/-WT, controls	1-way RM ANOVA	Within	D	6	42	9.935	<0.001
**Water intake**	GAL_2R-KO/-WT, DSS-treated	1-way RM ANOVA	Within	D	1.710	11.967	21.654	<0.001

Legend:
[1]RM: repeated measures
[2]D: Days
[3]G: Genotype
[4]T: Treatment

SI references

1 Einstein, E. B., Asaka, Y., Yeckel, M. F., Higley, M. J. & Picciotto, M. R. Galanin-induced decreases in nucleus accumbens/striatum excitatory postsynaptic potentials and morphine conditioned place preference require both galanin receptor 1 and galanin receptor 2. *Eur J Neurosci* **37**, 1541-1549, doi:10.1111/ejn.12151 (2013).

2 Brunner, S. M. *et al.* GAL3 receptor KO mice exhibit an anxiety-like phenotype. *Proc Natl Acad Sci U S A* **111**, 7138-7143, doi:10.1073/pnas.1318066111 (2014).

3 Hobson, S. A., Holmes, F. E., Kerr, N. C., Pope, R. J. & Wynick, D. Mice deficient for galanin receptor 2 have decreased neurite outgrowth from adult sensory neurons and impaired pain-like behaviour. *J Neurochem* **99**, 1000-1010, doi:10.1111/j.1471-4159.2006.04143.x (2006).

4 Oliveira, L. G. *et al.* Positive correlation between disease activity index and matrix metalloproteinases activity in a rat model of colitis. *Arq Gastroenterol* **51**, 107-112, doi:10.1590/s0004-28032014000200007 (2014).

5 Reichmann, F. *et al.* Dextran sulfate sodium-induced colitis alters stress-associated behaviour and neuropeptide gene expression in the amygdala-hippocampus network of mice. *Sci Rep* **5**, 9970, doi:10.1038/srep09970 (2015).

6 Erben, U. *et al.* A guide to histomorphological evaluation of intestinal inflammation in mouse models. *Int J Clin Exp Pathol* **7**, 4557-4576 (2014).

7 Reichmann, F., Painsipp, E. & Holzer, P. Environmental enrichment and gut inflammation modify stress-induced c-Fos expression in the mouse corticolimbic system. *PLoS One* **8**, e54811, doi:10.1371/journal.pone.0054811 (2013).

8 Brunner, S. M. *et al.* Validation of antibody-based tools for galanin research. *Peptides* **120**, 170009, doi:10.1016/j.peptides.2018.08.010 (2019).

9 Locker, F. *et al.* Lack of galanin receptor 3 alleviates psoriasis by altering vascularization, immune cell infiltration, and cytokine expression. *J Invest Dermatol* **138**, 199-207, doi:10.1016/j.jid.2017.08.015 (2018).
